# *Anopheles stephensi* [ә-nah′-fuhl-ēz ste′-fen-zī]

**DOI:** 10.3201/eid3204.241933

**Published:** 2026-04

**Authors:** Gaurav Kumar, Jaspreet Kaur

**Affiliations:** Indian Council of Medical Research National Institute of Malaria Research, Delhi, India

**Keywords:** Anopheles stephensi, mosquitoes, vector-borne infections, malaria

In 1901, George Michael James Giles, a lieutenant colonel and physician in the Indian Medical Service, described the *Anopheles stephensi* mosquito (Figure). He collected the mosquitoes from Ellichpur (currently Amravati district), India, and named the species in honor of John William Watson Stephens, a prominent British parasitologist who first described *Plasmodium ovale*, one of the human malaria parasites. Morphologically, *An. stephensi* mosquitoes can be identified by distinctive palpal ornamentation of equal apical and subapical pale bands and speckled appearance, pale and dark scales arranged along the veins of the wings, and dark and speckled pale bands on the hind tarsi.

**Figure:**
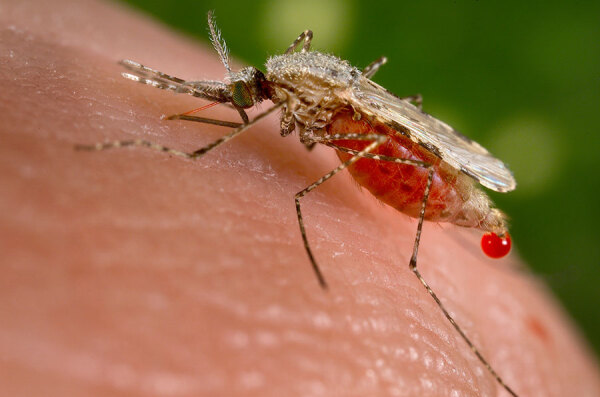
Female *Anopheles stephensi* mosquito. Photograph by Jim Gathany, from the Centers for Disease Control and Prevention Public Health Image Library.

*An. stephensi* (family Culicidae, subgenus *Cellia*) is an urban malaria vector responsible for ≈12% of malaria cases in India annually. After its detection in Africa in 2012, *An. stephensi* mosquitoes, if not controlled, are projected to put more than 126 million persons at risk for malaria.

## References

[1] Liston WG. A year’s experience of the habits of *Anopheles* in Ellichpur. The description of the species of *Anopheles* found in Ellichpur during the year. Ind Med Gaz. 1901;36:441–3. 29004095 PMC5164048

[2] Sharma VP. Current scenario of malaria in India. Parassitologia. 1999;41:349–53. 10697882

[3] Sinka ME, Pironon S, Massey NC, Longbottom J, Hemingway J, Moyes CL, et al. A new malaria vector in Africa: Predicting the expansion range of *Anopheles stephensi* and identifying the urban populations at risk. Proc Natl Acad Sci U S A. 2020;117:24900–8. 10.1073/pnas.2003976117 32929020 PMC7547157

[4] Subbarao SK, Vasantha K, Adak T, Sharma VP, Curtis CF. Egg-float ridge number in *Anopheles stephensi*: ecological variation and genetic analysis. Med Vet Entomol. 1987;1:265–71. 10.1111/j.1365-2915.1987.tb00353.x 2979540

[5] Sweet WC, Rao BA. Races of *A. stephensi* Liston, 1901. Ind Med Gaz. 1937;72:665–74. 29013249 PMC5173639

